# Impact of female age and male infertility on ovarian reserve markers to predict outcome of assisted reproduction technology cycles

**DOI:** 10.1186/1477-7827-7-100

**Published:** 2009-09-17

**Authors:** Tsung-Hsien Lee, Chung-Hsien Liu, Chun-Chia Huang, Kung-Chen Hsieh, Pi-Mei Lin, Maw-Sheng Lee

**Affiliations:** 1 Department of Obstetrics and Gynecology, Chung Shan Medical University Hospital, Taichung, Taiwan, Republic of China; 2 Institute of Medicine, Chung Shan Medical University, Taichung, Taiwan, Republic of China; 3 Department of Obstetrics and Gynecology, College of Medicine, National Taiwan University, Taipei, Taiwan, Republic of China; 4 Division of Infertility Clinic, Lee Women's Hospital, Taichung, Taiwan, Republic of China

## Abstract

**Background:**

This study was designed to assess the capability of ovarian reserve markers, including baseline FSH levels, baseline anti-Müllerian hormone (AMH) levels, and antral follicle count (AFC), as predictors of live births during IVF cycles, especially for infertile couples with advanced maternal age and/or male factors.

**Methods:**

A prospective cohort of 336 first IVF/ICSI cycles undergoing a long protocol with GnRH agonist was investigated. Patients with endocrine disorders or unilateral ovaries were excluded.

**Results:**

Among the ovarian reserve tests, AMH and age had a greater area under the receiving operating characteristic curve than FSH in predicting live births. Furthermore, AMH and age were the sole predictive factors of live births for women greater than or equal to 35 years of age; while AMH was the major determinant of live births for infertile couples with absence of male factors by multivariate logistic regression analysis. However, all the studied ovarain reserve tests were not preditive of live births for women < 35 years of age or infertile couples with male factors.

**Conclusion:**

The serum AMH levels were prognostic for pregnancy outcome for infertile couples with advanced female age or absence of male factors. The predictive capability of ovarian reserve tests is clearly influenced by the etiology of infertility.

## Background

Adequate follicular development of ovaries in response to gonadotropins has been referred to as ovarian reserve. The primary value of ovarian reserve markers is to provide assistance in selecting an appropriate protocol and/or initial dose of gonadotropins for controlled ovarian stimulation (COS) in IVF cycles. In addition to being makers for the ovarian response, an efficient indicator of pregnancy outcome prior to COS would be of enormous help during counseling, especially for expensive treatments, such as IVF and ICSI. As a result, markers for ovarian reserve or ovarian aging prior to COS are frequently utilized to predict the pregnancy outcome of IVF/ICSI cycles [1,2]. However, the biomarkers for ovarian reserve or ovarian aging prior to COS do not appear to predict pregnancy outcome efficiently [1,2].

Currently, the clinically used ovarian reserve markers include biochemical and sonographic markers. The serum FSH levels [3,4] and/or antral follicular counts (AFC) [5-7] in the early follicular phase represent the most common utilized biochemical and ultrasound markers for ovarian aging in clinical practice, respectively. Anti-Müllerian hormone (AMH), also referred to as Müllerian-inhibiting substance (MIS), is a member of the transforming growth factor β superfamily [8]. The granulosa cells within the preantral and small antral follicles in the ovaries are the sole source of AMH in rats [9] and humans [10]. The baseline serum AMH levels have a better capability in predicting the ovarian response to COS than other markers of ovarian reserve [11,12]. Indeed, the predictive value of AMH on pregnancy outcome has recently attracted the attention of clinicians. However, conflicting results regarding the correlation between the serum AMH level and pregnancy outcome have been reported in the literature [13-17].

The associated clinical factors for successful treatment of IVF/ICSI have been studied extensively [18-20]. The major clinical factors related to pregnancy outcome in IVF cycles include the following: age of the patient [18], embryo morphology [19], cause of infertility, and number of embryos transferred [20]. Among these factors, the age of the patients and the cause of infertility, together with the ovarian reserve markers, are available prior to COS in the general practice of IVF treatment.

The age of patients and the cause of infertility, however, are not independent factors regarding patients undergoing IVF/ICSI treatment. It has been reported that the prevalence of unexplained infertility increases in female patients of advanced age (> 35 years) seeking infertility treatment [21,22]. A putative cause of such unexplained infertility was attributed to diminished ovarian reserve. Nonetheless, the relationship between age and declining reproductive capacity is highly variable [23]. These data suggested that poor ovarian reserve may contribute to a significant proportion of unexplained infertility, especially for patients > 35 years of age. The ovarian reserve markers would probably connect with the outcome of pregnancy for such patients.

The ovarian reserve markers feature the reproductive function of the female partner of infertile couples without taking into consideration the effect of the male partner. Thus, the paternal effect is not evaluated by ovarian aging markers. Therefore, these markers may be more useful for couples with exclusive female factor infertility than couples with male infertility. Recently, van Rooij [24] reported that ovarian reserve tests are of limited value for predicting ongoing pregnancy in patients with mild male infertility and unexplained infertility. Their study further raises the possibility of an interaction between the etiology of infertility and the ovarian reserve tests regarding pregnancy outcome.

Herein we propose that the efficiency of ovarian reserve markers to predict pregnancy outcome may be better for advanced age women and infertile couples with exclusive female infertility. It would be helpful in clinical practice to clarify the effectiveness of ovarian reserve markers for specific subpopulations of patients seeking IVF/ICSI treatment. Therefore, we designed this study to compare the predictive power of various markers of ovarian reserve for the outcome of IVF/ICSI cycles using receiver operating curve (ROC) analysis.

Furthermore, the paternal effect is generally exhibited subsequent to fertilization. Embryo-related parameters may become dominant for predicting live births for infertile couples with male factor infertility. Consequently, embryo markers together with ovarian reserve markers and other clinical factors could be analyzed in a multivariable logistic regression model.

## Methods

### Patient selection and stimulation protocols

The initial survey for etiology of infertility included the following: computer-assisted semen analysis, hysterosalpingography or laparoscopy for tubal patency, and serum hormone levels of prolactin, estradiol, testosterone, FSH, LH, and TSH. All IVF/ICSI cycles performed at the Lee Women's Hospital in Taichung, Taiwan between March 2007 and December 2007 were enrolled. The patients with the following characteristics were included for analysis: a long protocol for the use of a GnRH agonist, first stimulation cycle for IVF/ICSI, the presence of bilateral ovaries, and the absence of endocrine disorders, such as polycystic ovarian syndrome or hyperprolactinemia. As a result, 336 IVF/ICSI procedures were analyzed for this study. The study protocol was approved by the Institutional Review Board of Chung-Shan Medical University Hospital. Consents were obtained from all the studied participants.

The presence of male infertility was determined according to the World Health Organization guidelines in 1999. Specifically, the semen analysis revealed results of normal (sperm concentration > 20×10^6^/ml, percentage of motile sperm > 50%, and normal sperm morphology ≥ 14%) for at least two of three separate repeat tests at the time of initial sperm evaluation, sperm storage, and the day of oocyte retrieval.

The women participating in this study followed a long protocol for the use of a GnRH agonist as that described in our previous report [25]. In brief, the protocol began with daily subcutaneous injections of 0.5 mg leuprolide acetate (LA Lupron; Takeda Pharmaceutics, Stolberg, Germany) on day 21 of the pre-stimulation cycle. Gonadotrophin (Gonal-F; Serono, Bari, Italy), at a dose of 225 IU/day subcutaneously, was administered for cycle days 3 -7 and then the dose was adjusted according to the ovarian response. The resulting ovarian response was monitored by transvaginal ultrasound and serum estradiol levels. When two or more follicles reached a maximum diameter of 18 mm, 250 μg of hCG (Ovitrelle; Serono) was administered. Transvaginal oocyte retrieval was performed 32-34 hours subsequent to hCG injection. Embryo transfer was performed three days after oocyte retrieval.

Serum hCG was checked 14 days subsequent to embryo transfer and patients with a level > 50 IU/L were considered pregnant. An ultrasound examination was preformed one week later and then again three weeks later in order to determine the number of intrauterine gestational sacs present and fetal viability, respectively. The visible fetal heart beat within theuterus by ultrasound constituted the definition of a clinical pregnancy. All the pregnant women subsequent to IVF/ICSI treatment were followed until the termination of pregnancy or live birth. A living child one week after delivery is defined as a live birth [26].

### Monitoring ovarian response and hormone assay

Baseline hormone profiles, including serum levels of estradiol, FSH, LH, and AMH were determined on day 3 of the pre-stimulation cycle. The AFC measurements were performed by the same technician on days 3-5 of the menstrual cycle. Antral follicles within the bilateral ovaries between two and ten mm in diameter were recorded. A 7.5 MHz transvaginal probe was utilized for all examinations. The ovarian response for all participants was monitored using transvaginal ultrasound from day 7 of the stimulation cycle and then continued at an interval of two days until the day of hCG administration.

Serum FSH and LH were measured using a specific immunometric assay kit (Immulite; Diagnostic Products Corporation, Los Angeles, CA, USA). The minimal detection limit for FSH was 0.1 IU/L and the intra- and inter-assay CVs for the FSH assay were 6.6% and 7.8%, respectively. The detection limit and intra- and inter-assay CVs for the LH assay were 0.1 IU/L and 6.5/7.1%, respectively. Estradiol and progesterone were also measured by competitive immunoassay using the Immulite kit, and the intra- and inter-assay CVs were 6.3% and 6.4% for estradiol and 6.3% and 5.8% for progesterone, respectively. The kit's minimal detection limits for estradiol was 15 pg/ml and 0.2 ng/ml for progesterone.

Measurement of serum AMH levels was performed using the AMH/MIS ELISA kit (Diagnostic Systems Lab, Webster, TX, USA). The minimal detection limit and intra- and inter-assay CVs for the AMH assay were 0.017 ng/ml and < 5% and < 8%, respectively.

### Classification of embryo quality

The embryos were classified according to the criteria proposed by Steer [27], as follows: grade 1, equally-sized blastomeres with no fragmentation; grade 2, equally- or unequally-sized blastomeres with < 20% overall fragmentation; grade 3, equally- orunequally-sized blastomeres with 20%-50% fragmentation; and grade 4, equally- or unequally-sized blastomeres with > 50% fragmentation. The embryos with < 20% overall fragmentation (grade 1 or 2), together with > 6 blastomeres on day 3 were considered as good embryos [28].

A previous report from our cooperative group suggested that transferring four embryos at the cleavage state would obtain the maximal pregnancy rate in IVF treatment [29]. In Taiwan, the IVF/ICSI treatment is not covered by health insurance; the financial burden has thus forced patients and clinicians to make an agreement for transferring a greater number of embryos. Therefore, the average number of transferred embryos would be approximately four to achieve a maximal pregnancy rate, even for the young age group, in this study. If there were ≤ 4 embryos available at the cleavage stage, then all embryos were transferred into the uterus.

### Statistical analysis

The various biologic parameters germane to IVF/ICSI cycles of the data were analyzed by the chi-square test or independent Student's t-test, as determined by respective conditions. The age strata utilized by the Centers for Disease Control and Prevention and the Society for Assisted Reproductive Technology for female age is as follows: < 35 years, 35 to < 38 years, 38 to < 40 years, and ≥ 40 years (Centers for Disease Control and Prevention, 2007). We attempted to identify the age groups adequate for clinical application of ovarian reserve markers; therefore, the youngest strata criterion (35 years) was utilized in this study.

ROC curve analysis was used to estimate the predictive power of the measured variables for pregnancy outcome. The relative ability of ovarian reserve tests to predict the IVF outcome were compared by calculating the areas under the ROC curve (ROC_AUC_) and the 95% confidence intervals (95% CIs). MedCalc software, version 9.3, was used to compare the areas under two ROC curves (MedCalc, Broekstraat, Belgium).

Since ovarian reserve markers only exhibit moderate predictive power for live births in IVF/ICSI cycles [1], additional parameters might be helpful in establishing a regression model for predicting outcome. In the literature, several parameters have been reported to be related to pregnancy outcome, such as etiology of infertility, duration of infertility, body mass index, the number of good embryos (NGE), and the number of transferred embryos (NET) [20]. A multivariate conditional logistical regression analysis was utilized with forward stepwise selection procedures. The selection of variables was based on a P-value < 0.2 to avoid erroneously excluding any prognostic factors [30]. A confidence level of P < 0.05 was considered to constitute the limit of statistical significance for comparison purposes. All the logistic regression analyses were performed by the Statistical Package for the Social Sciences, version 14.0 (SPSS Inc., Chicago, IL, USA).

## Results

Between March 2007 and December 2007, a total of 336 IVF/ICSI cycles were recruited for this analysis. We initially divided the patients into two groups by age (i.e., < 35 years and ≥ 35 years) and then calculated the difference for the biomarkers of ovarian reserve and etiology of infertility, as shown in Table 1. All the biomarkers of ovarian reserve surveyed (FSH, AFC, and AMH) showed a highly significant difference between young and advanced age patients (P < 0.001). The etiology of infertility also had significant differences between the young and advanced age groups of patients. The percentage of female factors unrelated to tubal factor increased (14.6% vs. 28.5%), whereas the incidence of male factors decreased (52.4% vs. 32.5%) for patients ≥ 35 years of age compared to the patients < 35 years of age (Table 1).

**Table 1:** Demographic data regarding the patients (n = 336) participating in IVF/ICSI treatment of different age groups.

	**Age < 35** **(n = 213)**	**Age ≥ 35** **(n = 123)**	**P value**
Chronologic age (years)	30.8 ± 0.2	38.6 ± 0.2	< 0.001^a^
Baseline FSH level (IU/L)	7.60 ± 0.26	9.63 ± 0.47	< 0.001^a^
Antral follicle count	7.8 ± 0.3	4.2 ± 0.3	< 0.001^a^
Baseline AMH level (ng/ml)	2.73 ± 0.13	1.85 ± 0.15	< 0.001^a^
Duration of infertility (years)	3.2 ± 0.2	4.7 ± 0.3	< 0.001^a^
Body mass index (kg/m^2^)	21.4 ± 0.2	22.0 ± 0.3	0.086^a^
Etiology of infertility			
Tubal factor	31 (14.6%)	22 (17.9%)	0.519^b^
Other female factor	31 (14.6%)	35 (28.5%)	0.003^b^
Male factor	111 (52.4%)	40 (32.5%)	< 0.001^b^
Mixed	34 (16.0%)	24 (19.5%)	0.504^b^
Unexplained	6 (2.8%)	2 (1.6%)	0.922^c^

The data are presented as the mean ± SD or percentage.

a. Comparison of the two groups by Student's t-test

b. Comparison by X^2 ^test

c. Comparison by Fischer's exact test

The outcome of IVF/ICSI cycles (i.e., the number of retrieved oocytes, the pregnancy rates, and the live birth rates) were all relevant to the chronologic age of the patients (P < 0.05, Table 2). In addition, the patients ≥ 35 years of age had higher doses of exogenous gonadotrophins, lower estradiol levels on the day of hCG administration, and a lower number of good embryos compared to the patients < 35 years of age. Unfortunately, the patients ≥ 35 years of age were characterized by a higher incidence of no embryos available for transfer than the patients < 35 years of age (5.8% vs. 0.5%, p = 0.004, Table 2). Furthermore, the implantation and live birth rates were lower for patients ≥ 35 years of age compared to those < 35 years of age (19.2% vs. 26.1%, p = 0.008 for implantation rate; 32.5% vs. 43.7%, p = 0.044 for live birth rate, Table 2).

**Table 2:** Outcome data regarding the patients (n = 336) participating in IVF/ICSI treatment of different age groups.

	**Age < 35** **(n = 213)**	**Age ≥ 35** **(n = 123)**	**P value**
Gonadotropin dose (IU)	1958 ± 23	2153 ± 45	< 0.001 ^a^
Estradiol levels on the day of hCG administration (pg/ml)	1976 ± 85	1391 ± 92	< 0.001^a^
Number of retrieved oocytes	12.3 ± 0.5	8.3 ± 0.5	< 0.001^a^
Number of transferred embryos	3.9 ± 0.1	3.1 ± 0.2	< 0.001^a^
Number of good embryos	3.2 ± 0.1	2.2 ± 0.2	< 0.001^a^
Cycle number with no oocytes retrieved	2/213 (0.9%)	2/123 (1.6%)	0.626^b^
Cycle number with no embryos transferred	1/211 (0.5%)	7/121 (5.8%)	0.004^b^
Pregnancy rate (%)	112/213 (52.6)	48/123 (39.0)	0.022^c^
Implantation rate (%)	217/830 (26.1)	74/386 (19.2)	0.008^c^
Multiple pregnancy rate (%)	64/112 (57.1)	20/48 (41.7)	0.106^c^
Live birth rate (%)	93/213 (43.7)	40/123 (32.5)	0.044^c^

The data are presented as the mean ± SD or percentage.

a. Comparison of the two groups by Student's t-test

b. Comparison by Fisher's exact test

c. Comparison by X^2 ^test

**Figure 1:**
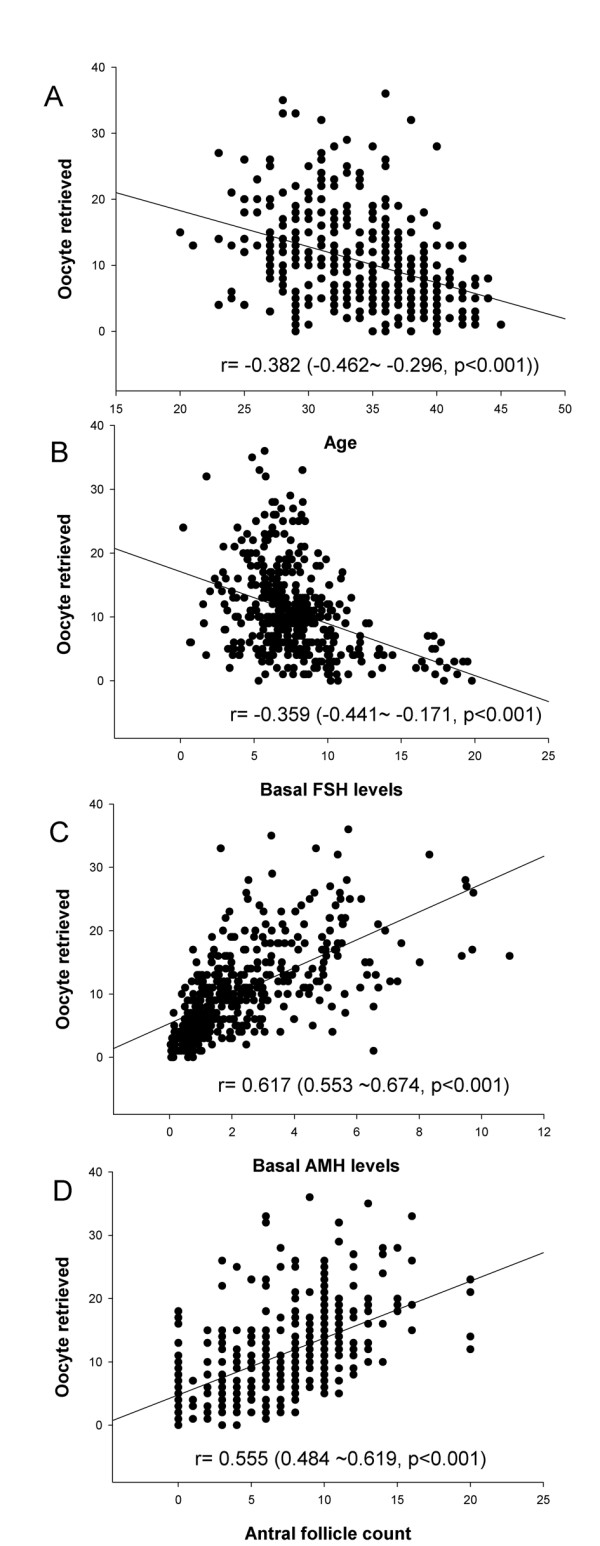
**The correlation between ovarian reserve markers and the number of retrieved oocytes**. A. chronologic age, B. basal FSH levels, C. basal AMH levels, D. antral follicle count. The Pearson correlation coefficient (r) was presented as the value (95% confidence interval, P value).

The number of retrieved oocytes was the primary outcome of ovarian response to COS in IVF/ICSI cycles in this study. We evaluated the prognostic value of those ovarian reserve markers for the number of retrieved oocytes by the Pearson correlation coefficient (r). Figure 1 demonstrates the relationship between the number of retrieved oocytes and the biomarkers of ovarian reserve. All the ovarian reserve markers, including age, baseline FSH levels, baseline AMH levels, and AFC (r = -0.382, -0.359, 0.617, and 0.556, respectively, all P < 0.001, Figure 1) were closely correlated with the number of retrieved oocytes. The AMH and AFC exhibited a better correlation than age and FSH for predicting the number of oocytes retrieved (P < 0.05).

Twelve patients (four due to no oocytes retrieved and eight due to no embryos available for transfer) did not undergo embryo transfer in this study. These cases were excluded fromthe following ROC curve and multi-variant logistic regression analyses. In addition, the values of ovarian markers did not have a normal distribution and the log transformation was performed for age, FSH, AFC, and AMH.

The area under the corresponding ROC curves (ROC_AUC_) for those markers were compared for the live births subsequent to IVF/ICSI cycles with at least one embryo transferred (Table 3). The levels of all ovarian reserve markers did not predict the pregnancy outcome at a significant level for patients < 35 years of age since the 95% CIs of ROC_AUC _for the corresponding curves include 0.5 (Table 3), while for women ≥ 35 years of age the levels of AMH and female age demonstrated significantly better predicting capability than FSH (p < 0.05, Table 3).

**Table 3:** Comparison of areas under the ROC curves of predictors (ovarian reserve markers) to live births.

**Ovarian reserve markers**	**Age < 35** **(n = 210)**	**Age ≥ 35** **(n = 114)**	**Male factor presence** **(n = 203)**	**Male factor absence** **(n = 121)**	**Total** **(n = 324)**
_log_FSH	0.517(0.441~0.593)	0.531(0.448~0.613)^ab^	0.527(0.456~0.597)	0.603(0.510~0.690)	0.524(0.468~0.579)
_log_(AFC+1)	0.573(0.496~0.647)	0.583(0.499~0.663)	0.571(0.478~0.660)	0.574(0.503~0.643)	0.572(0.517~0.627)
_log_AMH	0.511(0.435~0.587)	0.653(0.571~0.729)^a^	0.539(0.468~0.609)	0.654(0.562~0.738)	0.577(0.521~0.631)
_log_Age	0.508(0.432~0.584)	0.656(0.574~0.732)^b^	0.539(0.468~0.609)	0.572(0.479~0.662)	0.549(0.493~0.604)

The patients were divided into different groups by age (35 years) and the presence of male factor infertility. The data were presented as the area under the curve and the 95% confidence interval.

a: p = 0.035, b: p = 0.050, by comparison of the area under the corresponding ROC curve

We further separately analyzed the significance of those ovarian reserve markers in predicting pregnancy outcome for couples with and without male factors (Table 3). The individual ROC_AUC _of the corresponding ovarian aging markers had no predictive power for infertile couples with the presence of male factors. Nonetheless, the levels of FSH, AFC, and AMH, but not female age, exhibited the capability of predicting live births for infertile couples without male factors (Table 3).

By a multivariate logistic regression analysis, we found that the number of good embryos (NGE) was the sole important predictor for pregnancy outcome in IVF cycles (odds ratio: 1.319, p < 0.05, Table 4) for women < 35 years of age; however, the ovarian reserve markers were not significant predictors for patients of this subgroup. In contrast, for women ≥ 35 years of age, the _log_AMH and _log_Age were the sole significant predictors of pregnancy outcome in IVF cycles (odds ratio: 2.013 and 0.001, respectively, p < 0.05, Table 4). The embryo-related parameters were not as effective as ovarian reserve markers to predict pregnancy outcome for women ≥ 35 years of age.

**Table 4:** Odds ratio subsequent to conditional logistic regression model based on the surveyed ovarian reserve markers and pregnancy-related parameters.

**Variables**	**Age < 35** **(n = 210)**	**Age ≥ 35** **(n = 114)**	**Male factor presence** **(n = 203)**	**Male factor absence** **(n = 121)**	**Total** **(n = 324)**
_log_AMH	--	2.055 (1.285~3.286)	--	2.120 (1.308~3.436)	1.580 (1.197~2.086)
_log_AGE	--	0.001 (0.000~0.053)	--	--	--
*NGE*	1.319 (1.128~1.543)	--	1.322 (1.094~1.598)	--	--

The factors for analysis included female age, duration of infertility, body mass index, logAMH, log AFC, log FSH, number of transferred embryos, and number of good embryos *(NGE)*. The final prediction models were listed below the table. The significant coefficients were shown as the 95% confidence interval.

For patients < 35 years of age: Fertility index (live birth) = -0.633 + 0.277 × NGE

For patients ≥ 35 years of age Fertility index (live birth) = 34.256 + 0.700 × logAMH - 9.664 × logAGE

For couples with male factor: Fertility index (live birth) = -1.587 + 0.279 × NGE

For couples without male factor: Fertility index (live birth) = -0.637 + 0.751 × logAMH

For all the couples in this study: Fertility index (live birth) = -0.667+ 0.457 × logAMH

The probability (p) of live births can be calculated through p = e^Fertility index^/(1+e^Fertility index^)

Interestingly, the _log_AMH was the sole factor to predict live birth (odds ratio: 2.120, p < 0.05) for infertile couples without male factors subsequent to the logistic regression analysis (Table 4). The NGE was the major predictors of pregnancy and live births for infertile couples with male factors (odds ratio: 1.322, p < 0.05; Table 4).

The ROC curve analysis demonstrated that the AMH was able to predict live births in IVF cycles for women ≥ 35 years of age. The cut-off point indicated by the ROC curve for women ≥ 35 years of age was at a level of 1.68 ng/ml. The sensitivity was 60.0%, the specificity was 66.2%, the positive predictive value was 49.0%, and the negative predictive value was 75.4%.

**Figure 2:**
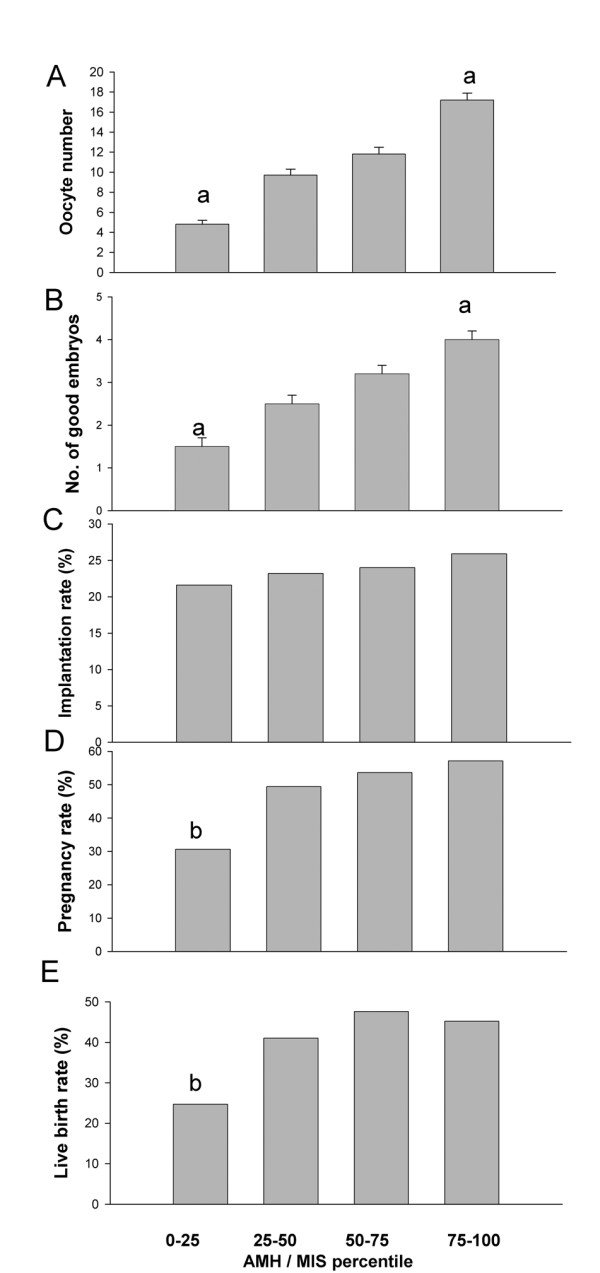
**Comparison of the outcome of IVF cycles grouping by baseline levels of serum anti-Mullerian hormone (the 25^th^, 50^th^, and 75^th ^percentile levels are 1.08 ng/ml, 1.97 ng/ml, and 3.26 ng/ml, respectively)**. A. The number of retrieved oocytes, B. the number of cultured good embryos, C. implantation rates, D. the rates of clinical pregnancy, E. the live birth rate. a. p < 0.05 by one-way ANOVA and *post-hoc *Bonferroni test compared to all the other three groups. b. p < 0.05 by X^2 ^test.

The baseline AMH values are not normally distributed, as described in previous reports [31,32], therefore the patients were classified into four subgroups according to the 25^th^, 50^th^, and 75^th ^percentile levels of serum AMH levels (1.08 ng/ml, 1.97 ng/ml, and 3.26 ng/ml, respectively) in order to clarify the predictive value of AMH for ovarian response and pregnancy outcome (Figure 2). The AMH levels were significantly correlated with the response to the number of retrieved oocytes and the number of cultured good embryos (Figures 2A and 2B). However, the AMH level was not significantly associated with the implantation rate (Figure 2C). The pregnancy and live birth rates for the patients in the lowest quartile group were significantly lower than the other three groups (Figures 2D and 2E).

## Discussion

In the present study, all the surveyed ovarian reserve markers, including baseline FSH levels, baseline AMH levels, and AFC, were significantly relevant to the chronologic age of the female partner of infertile couples (Table 1). Furthermore, all the ovarian reserve markers, including age, were intimately correlated with the number of oocytes retrieved (Figure 1). Specifically, AMH levels and AFC exhibited more prognostic value than age and FSH levels for ovarian reserve, i.e., the number of retrieved oocytes according to the 95% CI of the corresponding Pearson correlation coefficient. The results are concordant with previous reports [11,15]. Both the serum hormone levels (AMH) and ultrasound criteria (AFC) could be utilized for assessment of ovarian reserve at a similar level of significance.

The etiology of infertility is not independent from the age of the female patient, as demonstrated in Table 1. During the years prior to the menopause, oocytes in the human ovaries undergo an accelerated rate of loss until the cohort of oocytes is nearly depleted. It has been reported that this accelerated loss is initiated when the total number of oocytes reaches approximately 25,000, a number or threshold reached in normal women at an age of 37-38 years [33,34]. If the female partner of infertile couples exceeds the age of 38 years, the etiology of infertility would be classified as female age factor instead of unexplained infertility in our infertility clinic. Since endocrine disorders of female partners were excluded in the present study, the couples with feamle age factor (former unexplained infertility) contributed a significant proportion of other female factors for patients ≥ 35 years of age.

To predict pregnancy outcome or live birth by ovarian reserve markers is a relative complicated process. Based upon the results of this study, we identified that the studied ovarian reserve markers exhibited a predictive capability for live births only in some specific subgroups of infertile couples, such as those with advanced female age and/or absence of male factor infertility (Table 3). Among the ovarian reserve makers surveyed in this study, female age and AMH demonstrated better predictive power for live births than FSH for women ≥ 35 years of age (Table 3). The results revealed in Table 3 are compatible with our hypothesis that the prognostic value of ovarian reserve markers for live births was greater for women of advanced age than that for young aged women.

The ovarian reserve markers targeted the evaluation of ovarian function for the female partner of infertile couples. Therefore, we further suggest that these markers were more important for infertile couples with exclusive female factors than those couples with male factors. Table 3 demonstrated that AMH, AFC, and FSH were able to predict pregnancy outcome of IVF cycles for infertile couples without male factors. In addition, the ROC_AUC _for AMH featured the greatest area among these biomarkers, although the difference did not reach statistical significance. Interestingly, none of these biomarkers was able to predict the live births after ART cycles for couples with male factor infertility.

Several parameters obtained during the process of the infertility work-up have been reported to be relevant to the pregnancy outcome for IVF cycles, such as the etiology of infertility, body mass index, and the duration of infertility [20]. Furthermore, embryo morphology and number of transferred embryos are viewed as important predictors of pregnancy outcome in IVF cycles [19,35]. As a consequence, these parameters were added in the analysis of the logistic regression model for pregnancy outcome of IVF/ICSI cycles. The multivariate logistic regression model further confirmed that number of good embryos (an embryo-related parameter) was the major predictor for women < 35 years of age and/or infertile couples with male factors. By contrast, AMH (an ovarian aging marker) was more prognostic than embryo-related parameters for women ≥ 35 years of age or infertile couples without male factors (Table 4).

A high multiple pregnancy rates were observed accompanied with the embryo transfer policy in this study (Table 2). The different policy for embryo transfer in this study and other reports provide at least a partial explanation about the conflicting results in the literature for AMH in predicting pregnancy outcome [13-17]. If the policy regarding embryo transfer changes, the significance of ovarian reserve markers might change as well.

First, when the maximal number of transferred embryos is limited to two, as reported by Smeenk et al. [16] and Lekamge et al. [17], the ovarian reserve markers would have less of an influence on the number of good quality embryos destined to be transferred. Figure 2 shows that the AMH level in the lowest quartile was associated with a limited number of good embryos and a poor pregnancy outcome. The results suggested that the number of good embryos might be influenced by the levels of AMH, especially for patients with poor ovarian reserve, when the upper limit for the number of transferred embryos was three or more [13,15]. But in the case of an embryo transfer number limited to two, most patients would have sufficient good embryos for transfer. Therefore, the influence of AMH (ovarian reserve marker) would decrease in such a situation.

Second, the number of good embryos was higher in patients with higher AMH levels for all study-recruited women (Figure 1). Thereafter, those cycles with high AMH levels would have more embryos for cryopreservation and would result in high cumulative pregnancy rates. It has been reported that for women ≤ 38 years of age with a basal FSH < 10 IU/L, the AMHlevels is positively correlated with the cumulative pregnancy outcome [17]. They also reported that the clinical pregnancy rates per transfer in fresh cycles were not different between high and low AMH groups [17].

Regarding the relationship between embryo quality and AMH, controversial data exist in the literature [13,16]. In this study, the basal AMH level was not correlated with the implantation rate (Figure 1C). The results suggest that the association of AMH levels with the number of good embryos resulted from the fact that more oocytes were retrieved in IVF cycles from the patients with higher AMH levels. These results were similar to the finding reported by Hazout et al. in 2004 [13] and Eldar-Geva et al. in 2005 [15].

The range of AMH levels in this study were similar to that reported by Smeenk et al. [16], although the kit for AMH measurement was not the same in our study. The kit for AMH measurement was the same in those reports with conflicting results about AMH and pregnancy [13-17]. Furthermore, a recent study reported that the two kits for measuring AMH were closely correlated [36]. Therefore, the difference in ELISA kits for AMH do not account for the different results among the current study and those reports. In addition, the distribution of quartile levels in this study (1.08 ng/ml, 1.97 ng/ml, and 3.26 ng/ml) are similar to a previous report (5.7 pmol/l, 9.3 pmol/l, 16.4 pmol/l [for AMH levels, pmol/l = ng/ml × 7.143]) [32]. The current study and the report by Nelson et al. in 2007 [32] utilized the same kit for AMH measurement, which further supports the reproducibility of the AMH kit applied in the current report.

Our previous report [35] and the current study have both revealed that embryo-related parameters are not able to predict pregnancy outcome compared to biomarkers of ovarian reserve for women ≥ 35 years of age. The current study further supports the concept that an ovarian reserve evaluation is more important for women ≥ 35 years of age or infertile couples with exclusive female factors. These data will add greatly in counseling prior to COS, especially for women of advanced age or a relatively normal male partner subsequent to semen analysis, as demonstrated in Tables 3 and 4. The AMH levels might be utilized efficiently to discriminate diminished ovarian reserve for women ≥ 35 years of age, especially for couples with unexplained infertility [21].

## Conclusion

The ovarian reserve tests, especially AMH and AFC, are well-correlated to the ovarian response. When the results of ovarian reserve tests are utilized to predict the pregnancy outcome, the etiology of infertility and female age should be taken into consideration. The results of ovarian reserve tests, especially AMH, available prior to COS allows the clinician to give couples, for whom the female partner is ≥ 35 years of age or couples with exclusive female factors, a more accurate estimation for their chances of live births subsequent to IVF/ICSI treatment.

## Competing interests

The authors declare that they have no competing interests.

## Authors' contributions

LT participated in the design of the study, performed the statistical analysis and drafted the manuscript. LC participated in the design of the study and coordination and helped to draft the manuscript. HC carried out the immunoassays and helped to draft the manuscript. HK carried out the immunoassays. LP paticipated in acquisition of clinical data and helped to analysis and interpretation of data. LM conceived of the study and helped to revise the manuscript critically. All authors read and approved the final manuscript.
